# Time to RE-AIM: Why Community Weight Loss Programs Should Be Included in Academic Obesity Research

**DOI:** 10.5888/pcd13.150436

**Published:** 2016-03-17

**Authors:** Nia S. Mitchell, Allan V. Prochazka, Russell E. Glasgow

**Affiliations:** Author Affiliations: Allan V. Prochazka, Denver VA Medical Center, Denver, Colorado, and University of Colorado Anschutz Medical College, Aurora, Colorado; Russell E. Glasgow, University of Colorado Anschutz Medical Campus, Aurora, Colorado.

## Abstract

Despite decades of efficacy-based research on weight loss interventions, the obesity epidemic in the United States persists, especially in underserved populations. We used the RE-AIM (Reach, Efficacy/Effectiveness, Adoption, Implementation, and Maintenance) framework to describe the limitations of the current paradigm of efficacy-based research for weight loss interventions. We also used RE-AIM to propose that existing weight loss interventions (community-based programs) such as Jenny Craig, Take Off Pounds Sensibly (TOPS), and Weight Watchers be studied to supplement the efficacy-based research approaches to achieve population-level impact on obesity.

## Introduction

Despite the National Institutes of Health’s strategic plan to combat obesity and despite increased funding for obesity studies in recent years (1), the obesity epidemic persists. Almost 70% of US adults are overweight or obese, with highest rates among racial/ethnic minorities (2), low-socioeconomic–status groups (3), and rural populations (4). We attribute the lack of progress in reducing overweight and obesity to the current system, which favors efficacy-based studies of weight loss programs, that is, those that are proven in randomized controlled trials (RCTs). We suggest a parallel approach that includes studies that investigate existing community-based weight loss programs to augment the current system in order to have a larger effect on the obesity epidemic.

The purpose of this article is twofold: 1) to compare the efficacy-based and community-based approaches by using the RE-AIM (Reach, Efficacy/Effectiveness, Adoption, Implementation, and Maintenance) framework and 2) to propose that existing community-based weight loss programs (such as Jenny Craig, Take Off Pounds Sensibyle [TOPS], and Weight Watchers) be studied so they can supplement research-based programs (such as the Centers for Disease Control and Prevention’s National Diabetes Prevention Program) to combat the obesity epidemic. RE-AIM is used to evaluate interventions intended to have an impact on public health (5) and suggests that efficacious and effective programs will have minimal real-world impact if they have poor reach into target populations, inadequate adoption in different settings, insufficient implementation because of poor program fidelity, or minimal maintenance because of lack of sustainability. We used examples of efficacy-based approaches and community-based approaches. The National Diabetes Prevention Program (NDPP) is the example of an efficacy-based intervention. Jenny Craig, Weight Watchers, and TOPS, are examples of national commercial or nonprofit weight loss programs that could help achieve a population-level impact on obesity (Table) .

**Table:** Comparison of Community Weight Loss Programs in the United States, 2015

Characteristic	NDPP	Jenny Craig	Weight Watchers	TOPS
Year established	2010	1983	1963	1948
Financial structure	Varies^a^	Commercial^b^	Commercial^c^	Nonprofit^c^
Meeting format (weight loss)	Weekly	Weekly	Weekly	Weekly
Meeting format (weight maintenance)	Monthly	Monthly	At least monthly	Weekly
Leaders	Trained lifestyle coach	Jenny Craig consultant	Weight Watchers-certified coach	Volunteer peer leader
Meal plans	Variable	Meal replacement	PointsPlus^d^	Food Exchange program^e^ USDA MyPlate program^f^
Cost	$400 per year-long program	$15–$23 per day^g^	$240–$780 per year^h^	$92 per year

Abbreviations: NDDP, National Diabetes Prevention Program; TOPS: Take Off Pounds Sensibly; USDA, US Department of Agriculture.

a Payment varies. Some programs are free of charge (covered by grants, nonprofit organizations, or county funds); some insurers pay for all or part of the program; some participants pay the full cost.

b Some insurers may pay for part of the program. The program fees, but not meals, may be covered by some employers through flexible spending accounts, health savings accounts, or health reimbursement accounts.

c Some insurers may pay for all or part of the program.

d The PointsPlus system uses fiber, fat, protein, and carbohydrate content of food to assign a PointsPlus value to foods, and participants have a daily and weekly PointsPlus allowance. (Weight Watchers changed to the SmartsPoints system in December 2015. The new system uses the calories, saturated fat, sugar, and protein content of food to assign a SmartPoints value to foods, and participants have a daily and weekly SmartPoints allowance.)

e The Food Exchange System assigns exchanges in food categories (eg, starch, meat, fruit, vegetable, milk, fat) based on the daily calorie allowance.

f The USDA MyPlate program offers a graphic representation for portions of vegetables, fruits, proteins, grains, and dairy. It recommends the following fractional divisions of a plate: half fruits and vegetables, approximately one-fourth grains, approximately one-fourth protein, and a serving of low-fat or fat-free dairy.

g Jenny Craig offers 2 programs: All Access and As You Go. Daily food costs vary per participant. As participants get closer to their goal weight, they use fewer meal replacements and more of their own food. Therefore, their costs to Jenny Craig decrease.

h The cost of Weight Watchers varies by the membership option. OnlinePlus is $19.95 per month. Weekly in-person meetings are $44.95 per month. Personal coaching is $54.95 per month. All options include access to digital tools and applications.

### Efficacy-based program: National Diabetes Prevention Program

NDPP is a national program based on lessons learned from the Diabetes Prevention Program (DPP), an RCT of an intensive lifestyle intervention for overweight and obese people with prediabetes that encourages participants to lose 7% of their initial bodyweight and to increase physical activity to 150 minutes per week (6). Because weight loss and physical activity are essential components of all weight loss programs, we included NDPP in our evaluation. Unlike the DPP trial, which consisted of a one-on-one intervention with participants, NDPP is administered in group settings and requires that participants meet in weekly sessions for the first 6 months and then in monthly sessions for the next 6 months. NDPP comprises several components, including training and a registry. The training is coordinated through the Diabetes and Technical Assistance Center at Emory University and includes in-person sessions, webinars, and conference calls. The Diabetes Prevention Recognition Program is a registry of programs whose curricula and outcomes meet NDPP requirements for lifestyle-change programs, although they do not have to use DPP materials. Payment for the program is sometimes provided through partnerships with insurance providers, community organizations, (6) and government agencies so that participants do not necessarily bear the costs.

### Commercial program: Jenny Craig

Jenny Craig is a national commercial weight loss program that provides prepackaged meals to participants. The average daily calorie allowance is based on a daily deficit of 500 to 700 calories. The program recommends 150 minutes of activity per week for weight loss and 200 to 250 minutes of activity per week for weight maintenance. Participants get individual weekly sessions with a Jenny Craig counselor, either in person or by telephone. The program includes online tools for monitoring weight, activity, and calories (L. Talamini, RD, Jenny Craig, oral and written communications, December 2015).

### Commercial program: Weight Watchers

Weight Watchers is a national commercial weight loss program that uses a proprietary PointsPlus system, which assigns point values to foods. Values are based on the fat, carbohydrate, fiber, and protein content per serving. Participants are allowed a specific number of PointsPlus per day based on their height, weight, sex, and age. (In December 2015, Weight Watchers changed from the PointsPlus system to the SmartsPoints system. The new system uses the calories, saturated fat, sugar, and protein content of foods to assign a SmartPoints value to foods, and participants have a daily and weekly SmartPoints allowance.) Members achieve “lifetime” status once they reach and maintain their goal weight for 6 weeks and no longer pay membership fees as long as they do not exceed their goal weight by more than 2 pounds and get weighed once per calendar month. Participants choose their goal weight. However, they can only attain lifetime status if their goal weight corresponds to a body mass index (BMI) in the normal range (18.5 kg/m^2^–24.9 kg/m^2^), or they may choose a goal weight that corresponds to a BMI outside this range with a note from a health care provider. Weight Watchers offers 3 membership options, which all include online digital tools and applications: 1) OnlinePlus, 2) weekly in-person meetings, and 3) personal coaching. Weight Watchers also recommends increased physical activity. 

### Nonprofit program: Take Off Pounds Sensibly (TOPS)

TOPS is a national, nonprofit, peer-led weight loss program with a nationwide infrastructure. TOPS recommends the American Academy of Nutrition and Dietetics Food Exchange System and the US Department of Agriculture MyPlate program. The initial recommendation is that participants eat 20 to 25 calories per kilogram of actual body weight or an adjusted body weight while they are in the weight loss phase. TOPS recommends 150 minutes of moderate-intensity physical activity per week and requires that members consult a health care provider to determine a goal weight. When a participant reaches her goal weight, she becomes a KOPS (Keep Off Pounds Sensibly) member, which is the maintenance portion of the program, and she continues to attend weekly meetings.

## Evaluating Weight Loss Programs With RE-AIM

RE-AIM consists of 5 components: 1) reach, 2) efficacy/effectiveness, 3) adoption, 4) implementation, and 5) maintenance. NDPP, Jenny Craig, Weight Watchers, and TOPS were evaluated based on each component.

### Reach

Reach is the number or proportion of people who are willing and able to participate in an intervention (7). The initial reach of efficacy-based weight loss programs is limited because minimal infrastructure is available to support dissemination. For example, an efficacious weight loss intervention at one institution may not be available to someone who lives 100 miles away, and it can take years to develop the infrastructure necessary for widespread dissemination. The DPP results were published in 2002, but the NDPP was not authorized until 2010 (6). Although the NDPP currently has 751 programs listed on its website, only 41 have achieved full recognition as accredited programs; the rest are awaiting full recognition (8). National commercial and nonprofit programs may have a more extensive reach. For example, Jenny Craig was founded in 1983 and currently has 480 locations in the United States (9); TOPS was founded in 1948 and has 6,114 chapters in the United States (oral and written communications, M. Zouaghi, TOPS, July 2015). According to its website, Weight Watchers has more than 36,000 meetings worldwide each week, but it does not provide information about countries, states, cities, or locations. Additionally, all 3 programs have online tools, and Jenny Craig and Weight Watchers offer options for telephone counseling and coaching. Weight Watchers and TOPS have online plans, which further extend their reach. Because infrastructure already exists in these community-based programs, their reach can be broader than new efficacy-based programs, and thus community-based programs can have a broader impact.

### Efficacy/effectiveness

Efficacy or effectiveness is the impact (positive or negative) an intervention has on outcomes of interest (7). Efficacy is usually tested under ideal conditions in an RCT with strict protocols and rigorous inclusion and exclusion criteria to determine if an intervention has achieved the intended outcomes, with meticulous instructions for intervention delivery by trained study personnel, and with outcome measurements at study visits for which participants may be paid. Effectiveness is usually tested in real-world conditions, which may involve all eligible patients in a clinic receiving a modified version of an intervention (which may have been tested in an RCT) delivered by a medical assistant with follow up at routine clinic visits. Efficacy trials tend to have better outcomes than effectiveness trials.

The efficacy of RCT-based weight loss programs may be impressive. In the intensive lifestyle intervention of the DPP, adults with prediabetes were given a 16-week curriculum delivered in one-on-one sessions with subsequent monthly individual and group sessions. Half of participants achieved a goal weight loss of 7% or more at 24 weeks, and the intensive lifestyle group was 58% less likely to develop diabetes than the control group (10). NDPP is a modification of the DPP to make the program accessible to wider audiences by changing to a group format, adding facilitators with less formal training, and tailoring the program for specific groups (11).

The efficacy and effectiveness of Jenny Craig and Weight Watchers were also established in RCTs (12–18). One-year mean weight change in the Jenny Craig studies ranged from 7% to 11% (12,13), and in Weight Watchers studies, the mean weight change was approximately 5% to 6% (15–17). There were no efficacy studies of TOPS, but retrospective secondary database analyses of the program’s completers demonstrated its real-world effectiveness with an average weight loss of about 6%, clinically significant weight loss for approximately half of those who renewed their annual membership at one year, and weight loss maintenance for up to 3 and 7 years (19,20).

### Adoption

Adoption is the extent to which representative groups (eg, clinics, community groups) or individuals undertake an intervention (7). Efficacy RCT-based studies typically occur in settings that are not necessarily designed for broad population uptake. However, if interventions that were originally studied as efficacy-based RCTs are not widely adopted, they cannot have a population-level impact on obesity. Furthermore, the more training or equipment that is required to administer a program, the more difficult widespread adoption will be. Accordingly, it is difficult for low-resource groups, such as rural communities, to adopt efficacy RCT-based interventions delivered by specialists (eg, dieticians, nutritionists) or interventions that require resources such as costly fitness equipment.

NDPP training is coordinated through the Diabetes and Technical Assistance Center at Emory University and consists of in-person sessions, webinars, and conference calls. The Master Trainer Select program, which allows program graduates to train others, costs $1,500 per trainer for the initial training and $500 for the refresher course required to maintain certification status (21) (http://www.tacenters.emory.edu/news_events/news/MasterTrainerInstitute.html). Adoption of commercial programs varies because of geographic limitations, and such programs may not be available in low-income or rural areas that cannot sustain a commercial endeavor. TOPS is a peer-led program that does not require special expertise for its leaders; therefore, it has the potential to be adopted in low-resource settings.

### Implementation

The implementation of an intervention is the degree to which it is delivered as originally intended, which includes consistency of delivery, time, and cost (7). Consistency is also called program fidelity. The fidelity of efficacy-based weight loss interventions delivered in one-on-one or group settings may be high because of the quality of training the facilitators receive. However, these interventions may be impractical to deliver at community sites because of high costs. Even the NDPP costs about $400 per participant, although some insurers pay for the program (22).

Jenny Craig is delivered one-on-one, so it can be tailored for each individual, but the main components of the program may be delivered consistently to all participants. However, because it is a meal-replacement program, Jenny Craig can cost a participant $15 to $23 per day, which can cost hundreds to thousands of dollars annually, depending on how long participants remain in the program. Program costs, but not meal replacements, are sometimes covered by insurance. 

The Weight Watchers program is conducted by trained facilitators, and the same material is presented at all of its locations around the country each week; therefore, the fidelity is likely to be high. It can cost hundreds of dollars per year per participant, a portion of which may be covered by health insurance.

TOPS is also designed for group settings. TOPS allows individual chapters to adapt the intervention to fit their needs by choosing from more than 120 available programs. Each program has instructions about how it should be presented, but the quality of program delivery is not assessed. Therefore, fidelity to the intervention’s protocol probably varies among chapters. Its peer-led format keeps TOPS costs low for participants, about $92 annually, which makes it more accessible to low-income populations than more expensive programs.

### Maintenance

Maintenance is the sustainability of an intervention at individual and program levels (7). In weight loss interventions, the longer people remain in treatment, the longer they maintain prescribed behavior changes and sustain their weight loss (23). Maintenance of efficacy in RCT-based weight loss interventions can be limited because each is designed for a finite duration; therefore, it is difficult for participants to internalize behavior changes. The NDPP, our efficacy RCT-based example, is administered in 2 phases — a weight loss phase, which meets weekly for 6 months, and a maintenance phase, which meets monthly for 6 months. Commercial weight loss interventions, such as Weight Watchers and Jenny Craig, are designed with weekly contact during the weight loss phase and monthly contact during the maintenance phase. Regaining weight can occur when contact frequency decreases. In TOPS, people are expected to attend weekly meetings during the weight loss and maintenance phases, so weight regain may be attenuated.

## Discussion

Committing all research resources to development and study of the efficacy of new weight loss programs in RCTs would be unwise. From a public health standpoint, we must also focus on dissemination of existing programs with evidence of efficacy or effectiveness to reverse the obesity epidemic. As the Institute of Medicine stated in its report about preventing childhood obesity, “we need to use the best evidence available — as opposed to waiting for the best possible evidence” (24). Despite the many efficacious weight loss interventions based on RCTs, we have failed to make significant progress in treating obesity, especially for low-income and racial/ethnic minority populations. The reach of research focused on scaling up, repurposing, and redesigning efficacy-based weight loss interventions is limited for several reasons: infrastructure to support dissemination is minimal, widespread adoption is hindered by personnel requirements and high costs, implementation is jeopardized by the training requirements for leaders and lack of flexibility in content, and maintenance is limited because each intervention is designed for a finite duration, making it difficult for participants to internalize behavior changes.

To have a population-level impact on obesity, we must use weight loss interventions that have a national infrastructure. Programs should meet the following criteria: 1) have an extensive infrastructure that has a wide reach and the ability to be broadly disseminated, 2) be proven efficacious or effective, 3) be adoptable broadly in low-resource settings, 4) include curricula that can withstand various levels of implementation with low to moderate cost, and 5) be sustainable on individual and program levels.

The reach of Jenny Craig, Weight Watchers, and TOPS is superior to efficacy-based programs developed in research settings. Jenny Craig and Weight Watchers are efficacious programs, and TOPS is an effective program. Of these 3 programs, only TOPS is designed for low-resource settings. Jenny Craig and TOPS have flexible curricula that can withstand various levels of implementation, but the cost of participating in TOPS is more affordable for the general population. Weight Watchers has a less flexible curriculum, but it is implemented by trained employees in different settings across the country with high program fidelity, and it is moderately priced. TOPS started in 1948, Weight Watchers in 1963, and Jenny Craig in 1983, which indicates that all 3 programs are sustainable.

We do not suggest that academic institutions discontinue efficacy RCT-based obesity research. Many nonacademic weight loss interventions are based on information derived from RCTs. Of note, the Diabetes Prevention Recognition Program has a registry of programs whose curricula and outcomes meet its requirements but may not use specific DPP materials, and both Weight Watchers and Jenny Craig are pending recognition by the Diabetes Prevention Recognition Program, which indicates their curricula have been approved and evidence of outcomes is pending (8). TOPS plans to apply for recognition (oral communication, S. Luckey-Mueller,TOPS, December 2015). These developments further support our case that such commercial and nonprofit weight loss programs should be studied.

Development of new strategies to improve obesity management should continue. However, to have a population-level impact on obesity, funding agencies should also invest in studying existing programs that 1) have national infrastructure with a wide reach, 2) can be adopted broadly in low-resource settings, 3) have flexible curricula that can withstand variable levels of implementation with low to moderate cost, and 4) are sustainable.
